# Effect of cobalt-mediated Toll-like receptor 4 activation on inflammatory responses in endothelial cells

**DOI:** 10.18632/oncotarget.13260

**Published:** 2016-11-09

**Authors:** Sami A. Anjum, Helen Lawrence, James P. Holland, John A. Kirby, David J. Deehan, Alison J. Tyson

**Affiliations:** ^1^ Institute of Cellular Medicine, Newcastle University, Newcastle upon Tyne, UK; ^2^ Department of Orthopaedics, Freeman Hospital, Newcastle upon Tyne, UK; ^3^ Northern Retrieval Centre, Freeman Hospital, Newcastle upon Tyne, UK

**Keywords:** cobalt, TLR4, endothelium, inflammation, metal-on-metal, Immunology and Microbiology Section, Immune response, Immunity

## Abstract

Cobalt-containing metal-on-metal hip replacements are associated with adverse reactions to metal debris (ARMD), including inflammatory pseudotumours, osteolysis, and aseptic implant loosening. The exact cellular and molecular mechanisms leading to these responses are unknown. Cobaltions (Co^2+^) activate human Toll-like receptor 4 (TLR4), an innate immune receptor responsible for inflammatory responses to Gram negative bacterial lipopolysaccharide (LPS).

We investigated the effect of Co^2+^-mediated TLR4 activation on human microvascular endothelial cells (HMEC-1), focusing on the secretion of key inflammatory cytokines and expression of adhesion molecules. We also studied the role of TLR4 in Co^2+^-mediated adhesion molecule expression in MonoMac 6 macrophages.

We show that Co^2+^ increases secretion of inflammatory cytokines, including IL-6 and IL-8, in HMEC-1. The effects are TLR4-dependent as they can be prevented with a small molecule TLR4 antagonist. Increased TLR4-dependent expression of intercellular adhesion molecule 1 (*ICAM1*) was also observed in endothelial cells and macrophages. Furthermore, we demonstrate for the first time that Co^2+^ activation of TLR4 upregulates secretion of a soluble adhesion molecule, sICAM-1, in both endothelial cells and macrophages. Although sICAM-1 can be generated through activity of matrix metalloproteinase-9 (MMP-9), we did not find any changes in *MMP9* expression following Co^2+^ stimulation.

In summary we show that Co^2+^ can induce endothelial inflammation via activation of TLR4. We also identify a role for TLR4 in Co^2+^-mediated changes in adhesion molecule expression. Finally, sICAM-1 is a novel target for further investigation in ARMD studies.

## INTRODUCTION

Metal-on-metal (MoM) hip replacements are associated with the development of adverse reactions to metal debris (ARMD), which includes inflammatory pseudotumours, soft tissue necrosis, osteolysis and resulting aseptic implant loosening. Peri-implant tissues are often infiltrated by monocytes, macrophages and lymphocytes (referred to as aseptic lymphocyte-dominated vasculitis-associated lesion, ALVAL) which is indicative of an inflammatory response. However the cellular and molecular mechanisms that underlie ARMD are not well-understood.

Co^2+^ from MoM implants activates human Toll-like receptor 4 (TLR4) [1-3], an innate immune receptor expressed on immune cells as well as endothelial and epithelial cells. The major ligand for TLR4 is lipopolysaccharide from Gram negative bacteria, and receptor activation causes adaptor protein recruitment and an intracellular signalling cascade that upregulates the activity of transcription factors including NFκB [3].

We have previously shown that activation of TLR4 by Co^2+^ increases the secretion of inflammatory cytokines, including interleukin-8 (IL-8) and chemokine (C-X-C motif) ligand 10 (CXCL10), in MonoMac 6 macrophages [4]. Previous studies investigating the inflammatory effects of Co^2+^ in endothelial cells have primarily focused on endothelial cells transfected with TLR4 and its co-receptor MD2 [3, 5], but few studies have investigated the effect of Co^2+^ on endogenous TLR4-expressing endothelial cell lines. Endothelial cells are exposed to Co^2+^ present in the blood of MoM hip replacement patients [6] and therefore understanding the cellular response is important in defining the causes of ARMD and identifying potential therapeutic targets for ARMD prevention.

In the present study we assessed the immune response of endothelial cells to Co^2+^, with a focus on the role of TLR4. We also investigated the effect of Co^2+^ on adhesion molecule expression by endothelial cells and macrophages because of their critical role in inflammatory process such as leukocyte binding and extravasation.

## RESULTS

### Co^2+^ activation of TLR4 increases IL-8 and IL-6 secretion

**Figure 1:**
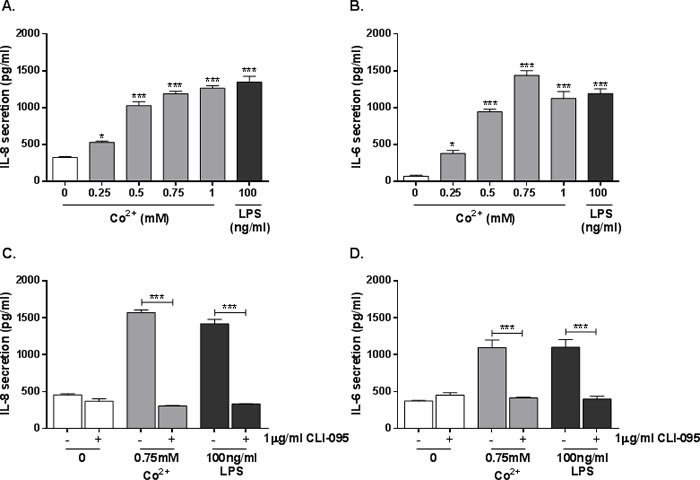
Effect of Co^2+^ ions and TLR4 activation on cytokine secretion by HMEC-1 **A.** & **B.** HMEC-1 were stimulated with 0.25-1mM Co^2+^ or 100ng/ml LPS. A. IL-8 and B. IL-6 secretion were assessed by ELISA. **C.** & **D.** HMEC-1 were pre-treated with 1μg/ml CLI-095 followed by 24h stimulation with 0.75mM Co^2+^ or 100ng/ml LPS. C. IL-8 and D. IL-6 secretion was quantified by ELISA. All data is representative of three independent experiments.

HMEC-1 cells were stimulated with 0.25-1mM cobalt chloride hexahydrate (Co^2+^) or 100ng/ml LPS for 24h and supernatant was collected for ELISA. IL-8 secretion was significantly increased by all concentrations of Co^2+^ (all *p* < 0.001 except 0.25mM where *p* = 0.026), peaking at 1300pg/ml with 1mM Co^2+^. The positive control LPS also increased IL-8 secretion (Figure 1A). IL-6 secretion was similarly upregulated by the agonists (all *p* < 0.001 except 0.25mM where *p* = 0.011) (Figure 1B).

To assess the role of TLR4 in the observed cytokine secretion, HMEC-1 were pre-incubated with 1μg/ml CLI-095 (a small molecule TLR4 antagonist) for 6h followed by stimulation with 0.75mM Co^2+^ or 100ng/ml LPS for 24h. IL-8 and IL-6 secretion were measured by ELISA. Pre-treatment with CLI-095 significantly decreased secretion of both cytokines in response to Co^2+^ (*p* < 0.001), showing that their release is TLR4-dependent. The cytokine release was not a result of Co^2+^-mediated cytotoxicity as trypan blue staining revealed no change in HMEC-1 viability following cobalt stimulation (Supplementary Material, Figure 6).

### Co^2+^-mediated TLR4 activation increases ICAM1 expression in endothelial cells and macrophages

Endothelial cells are known to express adhesion molecules, including intercellular adhesion molecule-1 (ICAM-1) and vascular adhesion molecule-1 (VCAM-1), which are essential in leukocyte extravasation in inflammation. We assessed the effect of Co^2+^ activation of TLR4 on *ICAM1* expression in HMEC-1 and MonoMac 6 macrophages. Macrophages also express CAMs for cell-cell communication.

**Figure 2:**
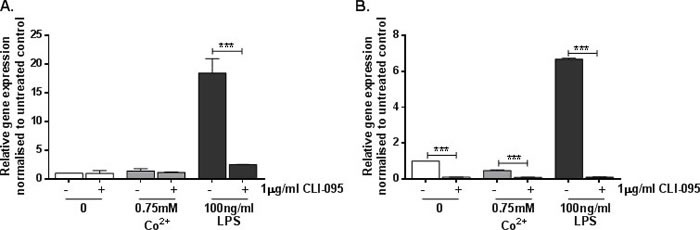
Effect of Co^2+^ and TLR4 activation on ICAM1 expression **A.** HMEC-1 and **B.** MonoMac 6 cells were stimulated with 1μg/ml CLI-095 for 6h prior to 24h stimulation with 0.75mM Co^2+^ or 100ng/ml LPS. RNA was isolated and cDNA synthesised by reverse transcription. *ICAM1* expression was quantified by qRT-PCR. Data is representative of three independent experiments.

Co^2+^ induced a small but significant 3-fold upregulation in *ICAM1* expression by HMEC-1 (*p* = 0.013) (Figure 2A) and a larger 35-fold upregulation in MonoMac 6 cells (*p* < 0.001) (Figure 2B). In both cell lines the response was found to be TLR4-dependent because it was inhibited by the TLR4 antagonist CLI-095 (both *p* < 0.001).

### Co^2+^ increases secretion of sICAM-1 in a TLR4-dependent manner

**Figure 3:**
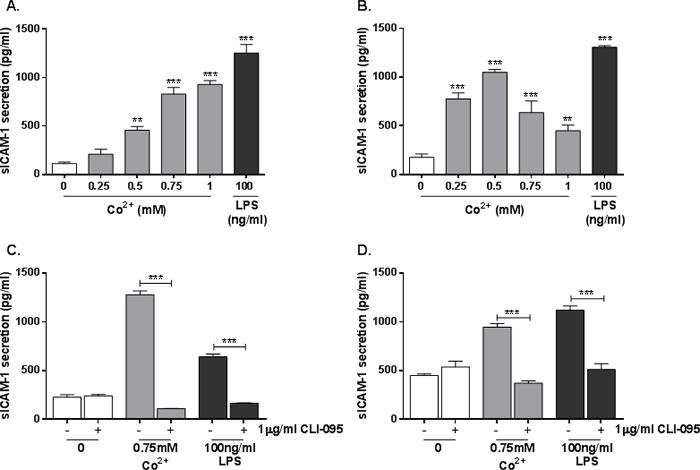
Effect of Co^2+^ and TLR4 activation on sICAM-1 secretion **A.** HMEC-1 or **B.** MonoMac 6 cells were stimulated with 0.25-1mM Co^2+^ for 24h and sICAM-1 secretion was measured by ELISA. **C.** HMEC-1 or **D.** MonoMac 6 cells were pre-incubated with 1μg/ml CLI-095 for 6h before stimulation with 0.75mM Co^2+^ or 100ng/ml LPS. All data is representative of three independent experiments.

In addition to its membrane-bound form, ICAM-1 can also be secreted as soluble ICAM-1 (sICAM-1). Given the TLR4-dependent increase in *ICAM1* expression described in Figure 2, we hypothesised that sICAM-1 release would also be affected by Co^2+^. Secretion of sICAM-1 by stimulated HMEC-1 and MonoMac 6 cells was investigated by ELISA. Cells were stimulated with 0.25-1mM Co^2+^ or 100ng/ml LPS for 24h. In HMEC-1, sICAM-1 secretion was increased by Co^2+^ concentrations of 0.5mM and above (all *p* < 0.001), peaking at 900pg/ml with 1mM Co^2+^ stimulation (Figure 3A). LPS increased sICAM-1 release to more than 1000pg/ml (*p* < 0.001). In MonoMac 6 cells sICAM-1 release was elevated across all Co^2+^ concentrations, peaking at 1000pg/ml with 0.5mM treatment (*p* < 0.001) (Figure 3B). As in HMEC-1, LPS elicited more sICAM-1 release than Co^2+^.

HMEC-1 and MonoMac 6 cells were then pre-incubated with 1μg/ml CLI-095 for 6h followed by 24h stimulation with either 0.75mM Co^2+^ or 100ng/ml LPS. There was a significant decrease in Co^2+^ and LPS-mediated sICAM-1 secretion in both HMEC-1 (Figure 3C) and MonoMac 6 cells (Figure 3D) (*p* < 0.001 in all cases). This shows that sICAM-1 release in response to Co^2+^ and LPS is TLR4-dependent.

### Co^2+^-mediated sICAM-1 secretion is not a result of MMP-9 activity

Previous studies have shown that sICAM-1 can be produced when mICAM-1 is cleaved by the gelatinase matrix metalloprotease-9 (MMP-9) [7]. LPS stimulates MMP-9 activity through activation of TLR4. We therefore investigated whether or not Co^2+^ activation of TLR4 also increases MMP-9 expression. HMEC-1 and MonoMac6 cells were pre-treated with 1μg/ml CLI-095 for 6h followed by 24h stimulation with 0.75mM Co^2+^ or 100ng/ml LPS. *MMP9* expression was assessed using qRT-PCR.

**Figure 4:**
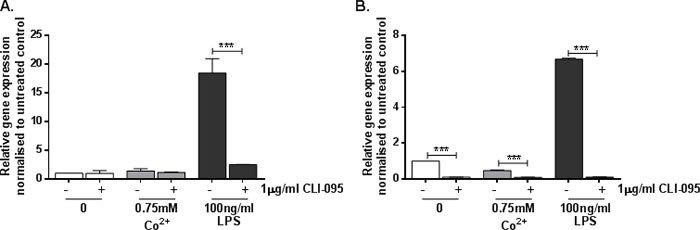
Effect of Co^2+^ and LPS on MMP9 expression **A.** HMEC-1 or **B.** MonoMac6 cells were pre-treated with 1μg/ml CLI-095 for 6h prior to stimulation with 0.75mM Co^2+^ or 100ng/ml LPS for 24h. *MMP9* expression was assessed by qRT-PCR. All data is representative of at least two independent experiments.

HMEC-1 exhibited a significant 16-fold increase in *MMP9* expression following stimulation with 100ng/ml LPS (*p* < 0.001) (Figure 4A). This was inhibited by CLI-095, showing that it is a TLR4-dependent effect (*p* < 0.001). In contrast there was no change in *MMP9* expression in response to Co^2+^ (*p* = 0.999) (Figure 4A). A similar pattern was observed in MonoMac 6 cells; following LPS stimulation there was a 7-fold increase in *MMP9* expression by (*p* < 0.001) (Figure 4B). CLI-095 inhibited this upregulated expression, showing that it is TLR4-dependent. However there was no increase in *MMP9* expression in response to Co^2+^, although CLI-095 decreased its expression further.

## DISCUSSION

In the present study we describe a TLR4-dependent inflammatory response to Co^2+^ in human endothelial cells and macrophages. HMEC-1 exhibited significant increases in secretion of inflammatory cytokines IL-8 and IL-6 when stimulated with Co^2+^. This was inhibited by the TLR4 antagonist CLI-095, showing that the receptor is central to the responses. Previous studies have shown that Co^2+^ upregulates adhesion molecule expression [8-10], but have not demonstrated the exact signalling pathways involved. The data obtained in this study supports the findings of these studies and also indicates a previously unidentified role for TLR4 in Co^2+^-mediated *ICAM1* expression in both endothelial cells and macrophages. Furthermore, for the first time a soluble adhesion molecule, sICAM-1, was detected in conditioned media from Co^2+^ and LPS-stimulated HMEC-1 and MonoMac 6 cells. CLI-095 inhibited sICAM-1 changes and consequently they are TLR4-dependent.

We investigated the effect of Co^2+^ on *MMP9* expression because MMP-9 can cleave membrane-bound ICAM-1 resulting in the release of its soluble form, sICAM-1. In addition, MMP-9 can be regulated by LPS activation of TLR4 [11] and therefore it is possible that Co^2+^-mediated TLR4 activation results in MMP-9 activity and sICAM-1 generation. However, although LPS increased *MMP9* expression in a TLR4-dependent manner, there was no change in expression in response to Co^2+^. The absence of any effect was consistent between HMEC-1 and MonoMac 6 cells. The lack of change in *MMP9* expression following Co^2+^ stimulation suggests that the enzyme is not responsible for the changes in sICAM-1 secretion observed in response to Co^2+^. Other proteolytic enzymes potentially involved in sICAM-1 cleavage include serine proteases [12], neutrophil elastase [13], and cathepsin G [14]. However the effect of Co^2+^ on these factors remains to be elucidated.

sICAM-1 has previously been proposed as a marker of inflammation [15] that is cleaved to regulate inflammatory responses but studies are now reporting a broader role for sICAM-1, including promotion of angiogenesis and neovascularisation [16]. This is of particular interest to the present study because blood vessel formation is required for pseudotumour development, which is a major factor in ARMD. Soft tissue necrosis is also a common feature of ARMD and can result from vascular inflammation restricting oxygen supply to the tissues. The ability of Co^2+^ to cause an inflammatory response, including pro-inflammatory cytokine release, in endothelial cells may indicate that similar effects occur *in vitro*, which could result in ischaemia and subsequent tissue death.

A limitation of the present study is the high Co^2+^ concentrations that we have used to stimulate the cells. Even the concentrations at the lower end of the range are considerably higher than those detected in the serum and synovial fluid of patients with failed MoM implants [17-19]. However, the Co^2+^ concentrations used in our study are in line with those of similar *in vitro* studies of the inflammatory effects of metal ions [3, 10, 20, 21]. Hence, they are appropriate and relevant for this study.

**Figure 5:**
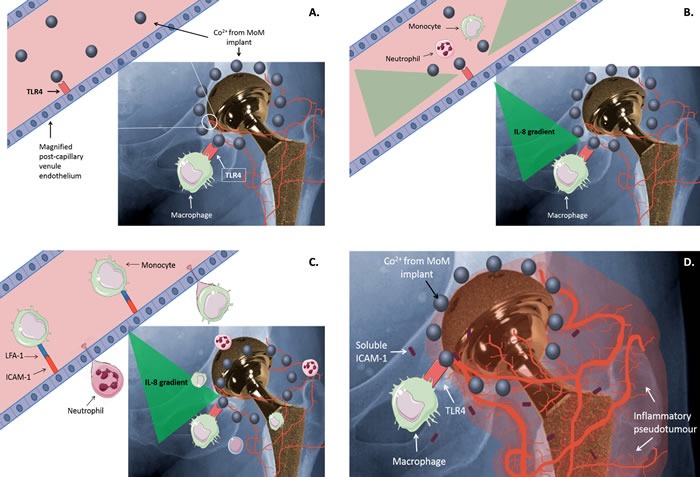
Working model **A.** Co^2+^ released from metal-on-metal (MoM) hip implants activates TLR4 on immune cells such as macrophages. **B.** Co^2+^-mediated TLR4 activation results in the release of inflammatory cytokines and chemokines, including IL-8, by macrophages and endothelial cells. The generated cytokine/chemokine gradient attracts circulating leukocytes, such as monocytes and neutrophils. **C.** Co^2+^ activation of TLR4 on endothelial cells increases expression of ICAM-1, which promotes leukocyte binding via interaction with LFA-1. This in turn drives leukocyte extravasation. **D.** Migrated leukocytes, together with sICAM-1 released by endothelial cells and macrophages, promote an inflammatory response that may contribute to inflammation and pseudotumour development around a MoM implant.

A working model of the possible mechanisms indicated by our results is shown in Figure 5. In summary, we have shown that Co^2+^ has the potential to induce an inflammatory response in the endothelium through activation of TLR4. It also shows for the first time that Co^2+^ increases sICAM-1 secretion in a TLR4-dependent manner. Although the exact mechanism of its release remains unclear, sICAM-1 is an interesting target for further investigation in ARMD because of its previously described roles in angiogenesis, neovascularisation and tumour formation [16].

## MATERIALS AND METHODS

### Cell culture

Human microvascular endothelial cells (HMEC-1) are derived from dermal foreskin. Cells were cultured in MCDB131 (Sigma-Aldrich, Gillingham, UK) medium supplemented with 10% foetal bovine serum (FBS), 50U/ml penicillin, 50μg/ml streptomycin, 10ng/ml epidermal growth factor (EGF) and 1μg/ml hydrocortisone (all Sigma-Aldrich).

MonoMac 6 cells are a human TLR4-expressing cell line derived from acute monocytic leukaemia. Cells were cultured as previously described [22].

### Cell stimulation

Cells were stimulated with cobalt chloride hexahydrate (referred to as Co^2+^ in this study) in complete culture medium appropriate for each cell line. Complete culture medium was used as a negative control while 100ng/ml TLR4-specific LPS (Alexis Biochemicals, San Diego, USA) provided a positive control.

### ELISA (IL-8, IL-6, sICAM-1)

Inflammatory cytokine secretion was quantified by enzyme-linked immunosorbent assay (ELISA). IL-6, IL-8 and sICAM-1 ELISA kits were purchased from Peprotech (London, UK) and assays performed as described previously [4].

### qRT-PCR

Gene expression changes were assessed by quantitative reverse transcriptase polymerase chain reaction (qRT-PCR) using TaqMan primers and probes (ThermoFisher Scientific, Massachusetts, USA). RNA was isolated using a Qiagen RNeasy Mini kit (Qiagen, Venlo, Netherlands) and cDNA synthesised using Superscript III reverse transcriptase (ThermoFisher Scientific). Each qRT-PCR reaction contained 5μl TaqMan Gene Expression Mastermix (ThermoFisher Scientific), 2μl diluted cDNA template, 2.5μl nuclease-free H_2_O and 0.5μl TaqMan Gene Expression Assay (ThermoFisher Scientific). No template controls with nuclease-free H_2_O instead of cDNA were included. All reactions were performed in triplicate and target gene expression was normalised to *GAPDH* expression.

### CLI-095

Inhibition of TLR4 was performed by pre-incubating cells for 6h with 1μg/ml CLI-095. CLI-095 (Invivogen, UK) is a small molecule TLR4 antagonist that binds to the intracellular domain of the receptor and prevents recruitment of downstream adaptor proteins.

### Cytotoxicity assay

Cytotoxicity was assessed by trypan blue staining. Stimulated cells were resuspended in a small volume of supernatant and 10μl cell suspension was mixed with 10μl trypan blue dye. Staining was visualised on a Luna II automated cell counter (Logos Biosystems, Virginia, USA)

### Statistical analysis

Statistical significance was calculated using a one-way analysis of variance (ANOVA). When samples were compared to an untreated control (Figures 1A, 1B, 3A, and 3B), Dunnett's test for multiple comparisons was performed. When comparing all samples to each other, Tukey's test for multiple comparisons was performed.

## SUPPLEMENTARY MATERIALS FIGURES


