# Supplementary material for: Effect of cobalt-mediated Toll-like receptor 4 activation on inflammatory responses in endothelial cells
Source: Oncotarget. 2016 Nov 9;7(47):76471–8. doi: 10.18632/oncotarget.13260 (PMC5363524; doi:10.18632/oncotarget.13260)
Supplement: Supplementary file 1 [file oncotarget-07-76471-s001.pdf]

Effect of cobalt-mediated Toll-like receptor 4 activation on inflammatory responses in endothelial cells

Supplementary Material

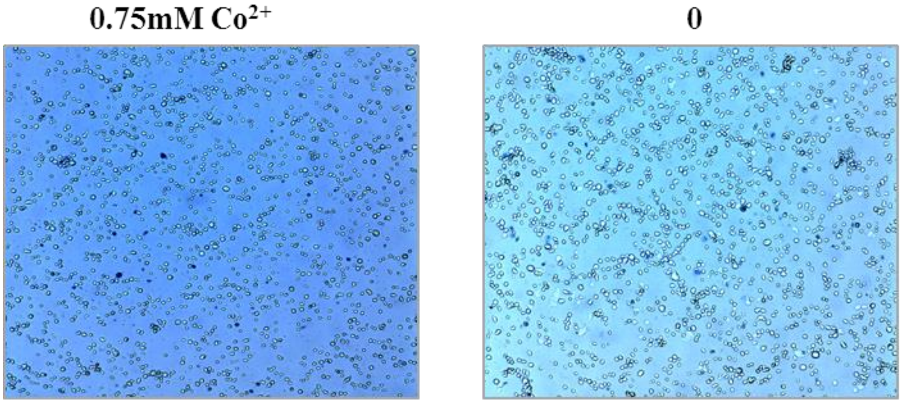

Figure 6: Effect of Co^{2+} on HMEC-1 viability

HMEC-1 cells were stimulated with 0.75mM Co^{2+} or left untreated for 24h and cell viability was assessed by trypan blue staining. Cell viability was normalised to 100% in the untreated cells. There was no change in viability in the Co^{2+}-stimulated cells.
